# Arabidopsis sucrose synthase localization indicates a primary role in sucrose translocation in phloem

**DOI:** 10.1093/jxb/erz539

**Published:** 2019-12-05

**Authors:** Danyu Yao, Eliana Gonzales-Vigil, Shawn D Mansfield

**Affiliations:** 1 Department of Wood Science, University of British Columbia, Vancouver, British Columbia, Canada; 2 University of Birmingham, UK

**Keywords:** Arabidopsis, carbon metabolism, cellulose, phloem loading, sucrose synthase

## Abstract

Sucrose synthase (SuSy) is one of two enzyme families capable of catalyzing the first degradative step in sucrose utilization. Several earlier studies examining SuSy mutants in Arabidopsis failed to identify obvious phenotypic abnormalities compared with wild-type plants in normal growth environments, and as such a functional role for SuSy in the previously proposed cellulose biosynthetic process remains unclear. Our study systematically evaluated the precise subcellular localization of all six isoforms of Arabidopsis SuSy via live-cell imaging. We showed that yellow fluorescent protein (YFP)-labeled SuSy1 and SuSy4 were expressed exclusively in phloem companion cells, and the *sus1/sus4* double mutant accumulated sucrose under hypoxic conditions. SuSy5 and SuSy6 were found to be parietally localized in sieve elements and restricted only to the cytoplasm. SuSy2 was present in the endosperm and embryo of developing seeds, and SuSy3 was localized to the embryo and leaf stomata. No single isoform of SuSy was detected in developing xylem tissue of elongating stem, the primary site of cellulose deposition in plants. SuSy1 and SuSy4 were also undetectable in the protoxylem tracheary elements, which were induced by the vascular-related transcription factor VND7 during secondary cell wall formation. These findings implicate SuSy in the biological events related to sucrose translocation in phloem.

## Teaser

Spatiotemporal localization of Arabidopsis sucrose synthase isoforms implicates these proteins in phloem loading and unloading.

## Introduction

In the vast majority of terrestrial plants, assimilated carbon from photosynthesis is primarily transported as sucrose to non-photosynthetic tissues, where it serves an integral role as a carbon and energy source. In sink tissues, sucrose is essential for the maintenance of cellular metabolism and cell wall biosynthesis, and can be converted to starch for storage and later use. Two enzymes catalyze the entry of sucrose into metabolic pathways: sucrose synthase (SuSy) and invertase (INV). While INV irreversibly cleaves sucrose into glucose and fructose, SuSy reversibly catalyzes the formation of fructose and UDP-glucose (Koch, 2004). SuSy predominantly occurs as a soluble form in the cytoplasm, but has also been found associated with the plasma membrane (Amor *et al.*, 1995).

Sucrose synthases have also been shown to play an important role in modulating sink strength (D’Aoust *et al.*, 1999). Phloem loading and sucrose breakdown efficiency in sink organs are thought to be important for defining sink strength. SuSy localization in phloem tissue has been observed through β-glucuronidase (GUS) histochemical staining or immunolabeling in many plant species, including maize (Nolte and Koch, 1993; Regmi *et al.*, 2016), potato (Fu and Park, 1995), rice (Shi *et al.*, 1994), citrus (Nolte and Koch, 1993), and Arabidopsis (Martin *et al.*, 1993; Fallahi *et al.*, 2008). In addition, several lines of evidence implicate SuSy in the development of sink organs. For example, inhibition of SuSy activity in potato led to decreased total tuber dry weight (Zrenner *et al.*, 1995), while in carrot plants suppression of SuSy expression resulted in shorter and thinner roots (Tang and Sturm, 1999). Along with being involved in phloem loading, SuSy has also been shown to participate in starch biosynthesis (Chourey *et al.*, 1998; Craig *et al.*, 1999), as cellular starch concentrations have been reported to be correlated with altered SuSy activity in potato leaves and tubers (Munoz *et al*., 2005; Baroja-Fernandez *et al*., 2009), tomato fruit (D’Aoust *et al.*, 1999), and maize seed endosperm (Li *et al.*, 2013).

In sink tissues, such as secondary xylem, SuSy has been proposed to have a specific role in the synthesis of cell wall polymers, including cellulose and callose (Amor *et al.*, 1995). A prevailing model of cellulose production suggests that a plasma membrane-localized SuSy interacts directly with the cellulose synthase complex to channel UDP-glucose derived from sucrose cleavage directly to cellulose synthesis (Koch, 2004; Verbancic *et al*., 2018). In developing cotton fibers, which deposit extremely pure cellulose in the secondary cell wall, Amor *et al.* (1995) showed via western blot analysis that SuSy was tightly bound to the plasma membrane. Consistent with this model, immunolabeling demonstrated that SuSy was abundant in sites where cellulose was rapidly synthesized and was distributed in a pattern paralleling the cellulose microfibrils (Amor *et al.*, 1995; Haigler *et al.*, 2001; Salnikov *et al.*, 2003). In addition, co-immunoprecipitation experiments conducted with developing *Populus* xylem showed that SuSy was pulled down with the cellulose synthase complex, among other known and putative cell wall-related proteins (Song *et al.*, 2010).

However, the essential role of SuSy in cellulose production has been questioned by Barratt *et al.* (2009), who reported that Arabidopsis *sus1/sus2/sus3/sus4* quadruple mutants lacking the majority of SuSy activity displayed, via Fourier transform infrared spectroscopy analysis, normal cell wall structure and cellulose content. This conclusion was later challenged by Baroja-Fernández *et al.* (2012), as they showed that the remaining SuSy5 and SuSy6 activity in the quadruple mutant was sufficient to support normal rates of starch and cellulose synthesis in Arabidopsis when measured under optimum conditions (Baroja-Fernandez *et al*., 2012). These results suggest that a high functional redundancy exists within the SuSy gene family. Moreover, mutant studies could not exclude the possibility that SuSy may directly facilitate cellulose biosynthesis, via the association with cellulose synthase complex. Therefore, this study employed high-resolution imaging of each SuSy isoform to provide additional information to understand the potential functionalities of SuSy in Arabidopsis development.

In Arabidopsis, xylem vessels and fiber cells form a thick secondary cell wall and serve as a major sink for cellulose deposition. If SuSy supplies the substrate to the cellulose synthase complex, one would expect to see SuSy isoforms in developing xylem cells as they are actively depositing cellulose polymers into their secondary cell walls. To investigate the potential role of SuSy in Arabidopsis growth and development, yellow fluorescent protein (YFP) fusion constructs were generated under the control of native SuSy promoters, and live-cell imaging was used to determine their precise intracellular localization. Both SuSy1 and SuSy4 were specifically localized to phloem companion cells, but were not detectable in the developing xylem of the organs examined, including stems, roots, petioles, and siliques. In addition, none of the other four SuSy isoforms was found in the developing xylem of elongating stem, indicating that Arabidopsis SuSy could not directly facilitate cellulose biosynthesis. Our data support a functional role for SuSy1 and SuSy4 in energy production in companion cells that could contribute to the intricacies of sucrose translocation.

## Materials and methods

### Plant material, growth conditions, and flooding treatment

The *sus1/sus4* double knock out mutant line was previously described in Bieniawska *et al.* (2007). Seeds of wild-type (WT) *Arabidopsis thaliana* ecotype Columbia-0 and *sus1/sus4* were sterilized with 70% (v/v) ethanol for 5 min, followed by 10% (v/v) sodium hypochlorite (bleach) for 15 min, and finally rinsed several times with sterile distilled water. After 2–4 d in the dark at 4 °C, seedlings were germinated on half-strength Murashige and Skoog (MS) medium without sucrose and transferred to soil 7 d post-germination. All plants were grown in a growth chamber maintained at 21 °C, 50% humidity, 16 h light, 8 h dark, and a photosynthetic photon flux density of 150–180 μmol m^–2^ s^–1^. After 4 weeks of growth, a subset of plants was subjected to flooding treatment by adding and maintaining degassed water in the growth trays at a level just above the surface of the soil for 5 d. Leaf diameters were measured at the widest part of the rosettes following the 5 d of flooding.

### Chlorophyll content analysis

The chlorophyll content index was measured on excised leaves, at the leaf tips where chlorosis was visually apparent by determining the absorbance at two wavelengths (931 nm and 653 nm) using a Chlorophyll Content Meter CCM-200 (OPTISCIENCE, USA). The output, the ratio of transmission of radiation from a light-emitting diode centered at 931 nm to transmission of radiation from a light-emitting diode centered at 653 nm (CCM-200 user manual), was used to establish the chlorophyll content index.

### Soluble carbohydrate analysis

Soluble sucrose was extracted as previously described (Coleman *et al.*, 2006) and analyzed by anion-exchange HPLC (ICS-5000; Dionex, Sunnyvale, CA, USA) that was fitted with a Dionex™ CarboPac™ PA1 column and a pulsed amperometric detector containing a gold electrode. Sugars were eluted with 16 mM NaOH and 2 mM NaOAc at a flow rate of 0.8 ml min^–1^. The concentration of sucrose was determined using an external calibration standard curve generated with known sucrose concentrations.

### Real-time quantitative PCR analysis

RNA was extracted from Arabidopsis stems and leaves using the TRIzol^®^ reagent (Invitrogen) according to the manufacturer’s instructions, and treated with TURBO DNase™ (Ambion) to remove residual DNA. cDNA was synthesized using the OneScript^®^ Plus cDNA Synthesis Kit (Applied Biological Materials), and real-time quantitative PCR was performed using a Bio-Rad CFX96 Touch Real-Time PCR Detection System following the manufacturer’s protocol. Three biological replicates were harvested for each treatment, and samples were run in triplicate with BrightGreen Express 2× qPCR MasterMix-No Dye (Applied Biological Materials) using the primers listed in Supplementary Table S1 at *JXB* online. The Arabidopsis *ubiquitin5* gene (*AtUBQ5*; At3g62250) was employed as a reference gene. The conditions for real-time analyses were: 95 °C for 20 s, followed by 40 cycles of 95 °C for 3 s, 57 °C for 30 s. Relative expression was determined according to Levy *et al.* (2004) using the equation: ΔCt=2^–(Ct*SuSy*–Ct*UBQ5*)^.

### Plasmid constructs

*SuSy* coding sequences were amplified from Arabidopsis stem and silique cDNA employing the iProof™ High-Fidelity PCR kit (Bio-Rad) using the primers listed in Supplementary Table S2. *SuSy* promoter fragments were amplified from genomic DNA, and the full details are listed in Supplementary Table S3. *SuSy1* and *SuSy4* coding sequences and individual promoters were first cloned into pDONR/Zeo using Gateway™ technology, and subsequently cloned into a modified pBin19 vector in which kanamycin resistance was replaced by a sulfadiazine resistance gene. C-terminal YFP fusions (*SuSy1*_*pro*_*::SuSy1*::*YFP* and *SuSy4*_*pro*_*::SuSy4*::*YFP*) were then independently transformed into *sus1/sus4* double mutant plants using *Agrobacterium tumefaciens* (strain GV3101). In the case of *SuSy2*, *SuSy3*, *SuSy5*, and *SuSy6*, all DNA sequences were cloned into pDONR/Zeo vectors and subcloned into pH7WG2 vectors containing YFP at the C-terminus and were driven by their respective native *SuSy* promoters (~2000 bp upstream of the start codon). All constructs were subsequently transformed into WT Col-0 plants.

### VND7 induction system for secondary cell wall formation

Seedlings containing both *35S*_*pro*_*::VND7::VP16::GR* and SuSy–YFP fusions (*SuSy1*_*pro*_*::SuSy1*::*YFP* and *SuSy4*_*pro*_*::SuSy4*::*YFP*) were crossed, selected, and germinated on germination medium (1× MS, 1% sucrose, 1× Gamborg’s Vitamin mix, 0.05% MES, 0.8% agar at pH 5.8) for 5 d. Dexamethasone at 10 μM (diluted in half-strength MS liquid solution, Sigma) was supplemented to the plates for 12 h (the earliest stage of secondary cell wall deposition) to 24 h to induce the differentiation of protoxylem tracheary elements (Yamaguchi *et al.*, 2010; Watanabe *et al.*, 2015). Induction time for each cell in the individual seedling was slightly different between plants and lines, so the stage of development was defined based on the morphology of secondary cell wall banding pattern, as per Watanabe *et al*. (2015, 2018).

### Western blotting

Approximately 200 mg of 6-week-old Arabidopsis stems were ground in liquid nitrogen and used for protein extraction following a previously described method (Wang *et al.*, 2006). Proteins were quantified using DC™ Protein Assay (Bio-Rad) and diluted in phosphate-buffered saline (PBS) containing 1% SDS. A 15–25 μg aliquot of total protein was loaded onto an 8% SDS–PAGE gel with a 5% stacking gel and separated for 120 min at 110 V using the Mini Protean^®^ Tetra Cell system (Bio-Rad). Proteins were subsequently transferred to a 0.1 μm pore nitrocellulose membrane and used for western blot analysis performed as previously described (Schilmiller *et al.*, 2007). Primary antibodies (Hill *et al.*, 2014; Watanabe *et al.*, 2018) were used at the dilutions of 1:2000 for anti-CesA4, 1:500 for anti-CesA8, and 1:250 for anti-CesA7 in Tris-buffered saline with 0.01% Tween-20 containing 1% non-fat dry milk. Anti-SuSy immune serum raised against soybean root nodule SuSy (Zhang et al., 1999; Fedosejevs et al., 2014) was used at a 1:5000 dilution. Blots were washed four times with Tris-buffered saline with 0.01% Tween-20 and then incubated with a Superclonal™ ECL horseradish peroxidase- (HRP) conjugated secondary antibody (goat anti-rabbit IgG, Thermo Fisher) at a concentration of 1:15 000. Protein–antibody complexes were visualized with the SuperSignal West Pico Chemiluminescent Substrate kit (Thermo Fisher) following the manufacturer’s instructions.

### Microscopy

Live-cell imaging was performed on a Leica DMI 6000 B inverted microscope with a Perkin Elmer Ultraview VoX Spinning Disk scan head with excitation/emission wavelengths of 514 nm/540 nm for visualization of all SuSy–YFP fusions. Propidium iodide and FM4-64 were detected using the 561 nm laser line and the 595 nm emission filter, respectively, while aniline blue was excited by a 405 nm laser and observed with the 540 nm detection filter. Images were captured on a Hamamatsu 9100-02 CCD camera using Volocity 6.3 software (Improvision) and processed using Image J software (National Institutes of Health; https://rsb.info.nih.gov/ij/). Background correction was performed by adjusting brightness or contrast of the images. For visualizing SuSy localization in mature plants, thin cross-sections were hand-sectioned from the base to the top of elongating stems and from leaves which were still expanding. In addition, 60–100 μm thick longitudinal sections of developing petioles and siliques were generated using a sliding benchtop microtome (American optical, Model #860). Cell wall staining with propidium iodide was performed by incubating 7-day-old seedlings in 10 μg ml^–1^ propidium iodide (in water) for 5 min, followed by thorough washing in water prior to imaging. The plasma membrane was labeled with a 10 μM FM4-64 dye (in water, Invitrogen) for 1 min and imaged directly, while the sieve plates were labeled using 0.01% aniline blue (in water) for 10 min in the dark and then rinsed, prior to imaging.

## Results

### Phenotype of *sus1/sus4* under hypoxic conditions

**Fig. 1.**
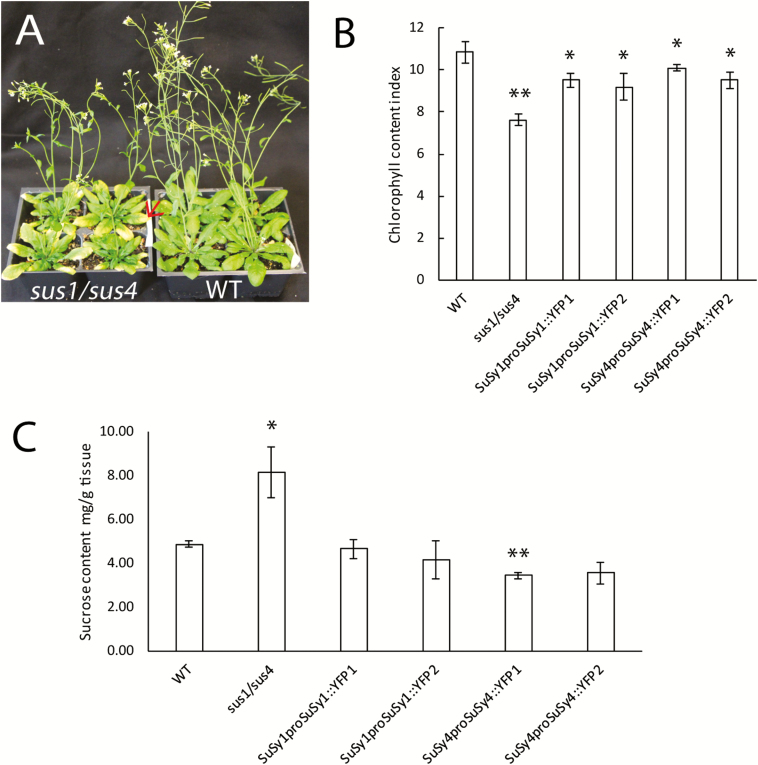
Phenotypes of *sus1/sus4* mutants and complemented Arabidopsis lines. (A) Representative photos of 6-week-old WT and *sus1/sus4* mutants grown in a growth chamber maintained at 21 °C, 50% humidity, 16 h light, 8 h dark, and a photosynthetic photon flux density of 150–180 μmol m^–2^ s^–1^, showing the leaf chlorosis (red arrow) in double mutant plants. (B) Chlorophyll content index of the rosette leaves of *sus1/sus4*, WT, and SuSy transgenic plants. Results represent the means ±SE of three replicates from each of four individual plants (*n*=4). (C) Leaf soluble sucrose content after 5 d of flooding. Data are means ±SE calculated from four individual plants per line. Asterisks indicate a significant difference (*P<0.05, **P<0.01) comparable with WT plants using a Benjamini–Hochberg-corrected Student *t*-test.

The Arabidopsis stem is considered a model system to study cell wall synthesis and, more specifically, cellulose biosynthesis, as it is largely composed of xylem vessels and fiber cells, which in turn are dominated by the cellulose polymer. In addition, *SuSy1* and *SuSy4* have been identified as the most highly expressed stem isoforms (Bieniawska *et al.*, 2007), and as such were selected to examine their putative contribution to cellulose synthesis and deposition. Consistent with Bieniawska *et al.* (2007), under normal growing conditions, the *sus1/sus4* double mutant exhibited no obvious growth phenotypes compared with WT plants, except for the prevalence of chlorotic leaves (Fig. 1A). In contrast, when subjected to 5 d of flooding, these lines showed reduced growth rates and significantly elevated soluble sucrose content in the rosette leaves (Fig. 1C; Supplementary Table S4).

### SuSy1 and SuSy4 are detected in phloem, but not in developing xylem

**Fig. 2.**
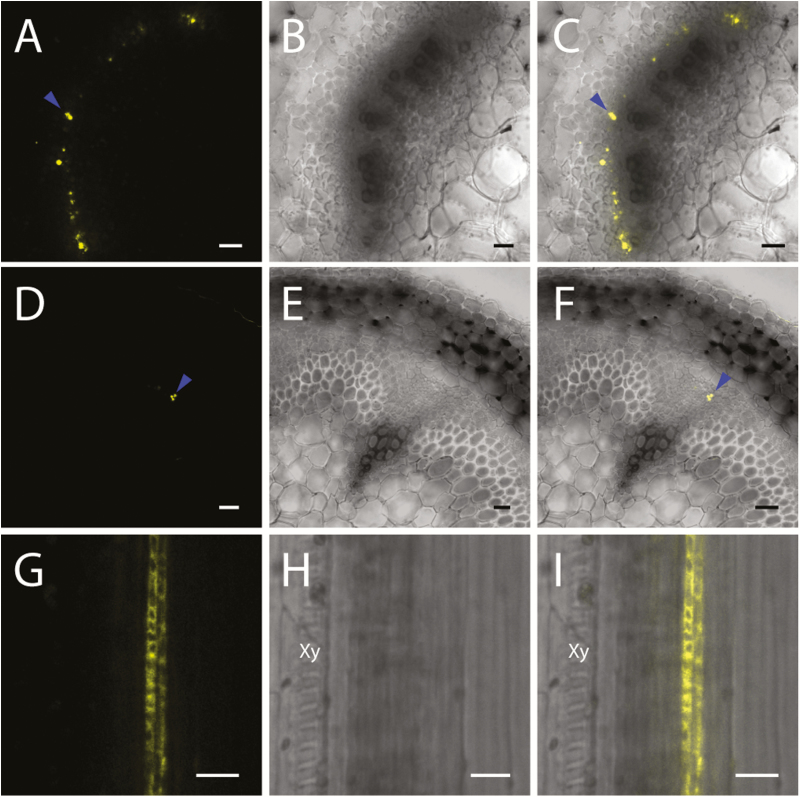
Confocal images of AtSuSy1–YFP in aerial tissues. (A–C) SuSy1–YFP localization in the phloem of stem cross-sections. (D–F) Cross-sections of leaf petioles showing YFP signal of AtSuSy1 (blue arrowheads) in the phloem. (G–I) Longitudinal sections of silique walls showing SuSy1–YFP in phloem tissue, not in protoxylem. Xy, protoxylem; (A), (D), and (G) are YFP fluorescence panels, while (B), (E), and (H) represent bright field images, and (C), (F), and (I) are merged images of YFP fluorescence and bright field images. Scale bars=50 μm in (A–F) and 20 μm in (G–I).

To investigate the spatiotemporal localization of these isoforms, Arabidopsis *SuSy1* (At5g20830) and *SuSy4* (At3g43190) coding sequences were cloned and subsequently fused to a C-terminal YFP fluorescence tag. The constructs were placed under the regulation of their respective native *AtSuSy1* or *AtSuSy4* promoters, and the functionality of the SuSy–YFP proteins was tested by complementing the *sus1/sus4* loss-of-function mutant. In general, individual SuSy–YFP fusions partially rescued the *sus1/sus4* phenotypes, demonstrating partial recovery of leaf chlorosis (Fig. 1B) and a reduced growth rate (Supplementary Table S4), and complete recovery of the soluble sucrose concentrations under hypoxic conditions (Fig. 1C). Subsequently, live-cell imaging was performed to visualize the cellular distribution of SuSy1 and SuSy4 proteins via spinning disk confocal microscopy. By checking the cross-sections from the base to the top of an elongating stem, YFP signal was not detectable in the xylem, but was consistently shown to be abundantly present in phloem tissue (Fig. 2A–C; Supplementary Fig. S1A–C). The same distribution pattern was also observed in the petioles of expanding leaves and developing siliques (Fig. 2D–I; Supplementary Fig. S1D–I). In the roots of 7-day-old seedlings, protoxylem cells were stained with propidium iodide and the fluorescently tagged SuSy signal was observed specifically in the phloem poles (Supplementary Fig. S2). SuSy1 and SuSy4 were also found in the funiculus, and only SuSy1 was present in the unloading domain of seeds at the end of the funiculus (Supplementary Fig. S3).

### SuSy1 and SuSy4 are confined to companion cells

**Fig. 3.**
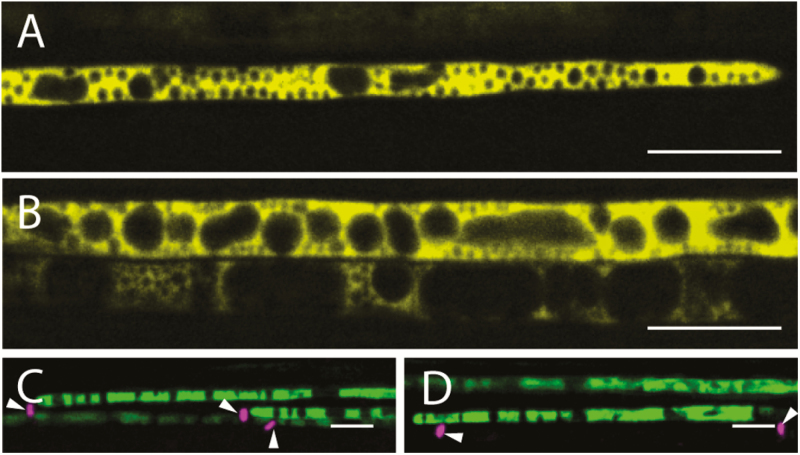
AtSuSy1–YFP and AtSuSy4–YFP were found specifically in the companion cells of petioles. (A) Longitudinal sections of leaf petioles showing AtSuSy1 in companion cells containing numerous intracellular organelles. (B) AtSuSy4–YFP was confined to companion cells of leaf petioles. (C) Associated sieve plates were stained with aniline blue (magenta; white arrowheads) in panels showing YFP signal of AtSuSy1 (green). (D) Sieve plates were labeled with aniline blue (magenta; white arrowheads) in panels showing YFP signal of AtSuSy4 (green). Scale bars=10 μm.

To further examine the precise localization of SuSy1 and SuSy4 proteins, thin longitudinal sections of leaf petioles that allowed single-cell imaging were employed. SuSy1 and SuSy4 were detected extensively in cells showing the typical morphology of companion cells (Fig. 3A, B), which differ from sieve elements as they inherently contain nuclei and vacuoles. To support this observation, the identity of these cells was confirmed by aniline blue staining, which specifically labels the callose-enriched sieve plates of sieve elements (Fig. 3C, D). Interestingly, both SuSy–YFP fusion proteins were consistently observed streaming within the cytoplasm of the companion cells, as shown by time-lapse video (Supplementary Movie S1).

### Secondary cell wall CesAs are expressed concurrently with the SuSy isoforms

**Fig. 4.**
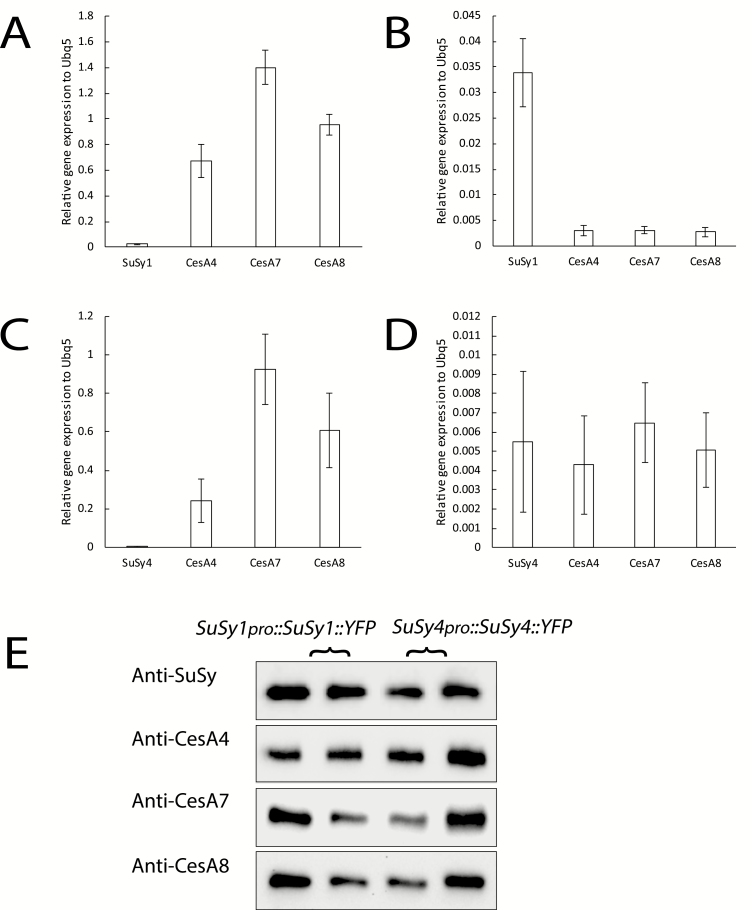
Transcript level and protein abundance of Arabidopsis secondary cell wall CesAs in 6-week-old SuSy1 and SuSy4 transgenic plant lines. (A, B) Expression of Arabidopsis *AtSuSy1*, *AtCesA4*, *AtCesA7*, and *AtCesA8* relative to *AtUBQ5* in the stems (A) and rosette leaves (B) of a SuSy1 transgenic line. (C, D) Expression of Arabidopsis *AtSuSy4*, *AtCesA4*, *AtCesA7*, and *AtCesA8* relative to *AtUBQ5* in the stems (C) and rosette leaves (D) of a SuSy4 transgenic line. (E) Western blots showing that SuSy–YFP and secondary cell wall CesA (CesA4, CesA7, and CesA8) proteins were all present in the stems of transgenic lines containing *SuSy1*_*pro*_*::SuSy1::YFP* or *SuSy4*_*pro*_*::SuSy4::YFP.* Data in expression bar graphs are means ±SE calculated from three biological replicates (*n*=3). For western blots, two biological replicates were used (*n*=2).

Cellulose synthesis will slow as the xylem matures. Should a SuSy–cellulose synthase complex interaction exist, SuSy–YFP fusion proteins may go undetected if xylem development has transitioned to later phases of development. To exclude this possibility, transcript levels and protein abundances of secondary cell wall-specific CesAs (CesA4, CesA7, and CesA8) were examined in the same transgenic SuSy1 and SuSy4 lines used for live-cell imaging. In general, CesA4, CesA7, and CesA8 displayed extremely high expression levels in elongating stem tissues but not in developing rosette leaves (Fig. 4A–D), which is consistent with their biological roles in secondary cell wall cellulose synthesis. Moreover, the transcript abundance of the secondary cell wall CesAs was much higher than that of SuSy in the stem tissue of both SuSy1–YFP and SuSy4–YFP fusion lines (Fig. 4A, C). Furthermore, western blot analysis showed that the CesA4, CesA7, and CesA8 proteins were present in the examined elongating stem tissues (Fig. 4E), indicating that live-cell imaging was carried out at an appropriate developmental window when secondary cell walls were still being actively deposited.

### SuSy1 and SuSy4 are not present in the VND7-induced tracheary element system

In 2005, Kubo *et al.* (2005) demonstrated that the overexpression of Arabidopsis VND7 transcription factor could induce the trans-differentiation of seedling cells into protoxylem tracheary elements. The VND7 transcription factor was later paired with an inducible transcriptional activator, the viral protein 16 (VP16) coupled with a glucocorticoid receptor (GR), and, upon induction with dexamethasone, even the epidermal cells in Arabidopsis seedlings were shown to differentiate into tracheary elements (Yamaguchi *et al.*, 2010). This system has subsequently been used to successfully perform high-resolution imaging of proteins expressed during secondary cell wall deposition (Watanabe *et al.*, 2015, 2018; Takenaka *et al.*, 2018). SuSy1–YFP and SuSy4–YFP fusions were transformed into an Arabidopsis line carrying the VND7–VP16–GR insertion and the ensuing plants showed YFP signal confined to the vasculature of seedling roots (Supplementary Fig. S2). However, the YFP signal of either SuSy1 or SuSy4 was undetectable in the induced tracheary element cells over the course of secondary cell wall development (Supplementary Fig. S4), indicating that SuSy1 and SuSy4 are not concurrently up-regulated during secondary cell wall formation in protoxylem tracheary elements.

### SuSy5 and SuSy6 are restricted to sieve elements

**Fig. 5.**
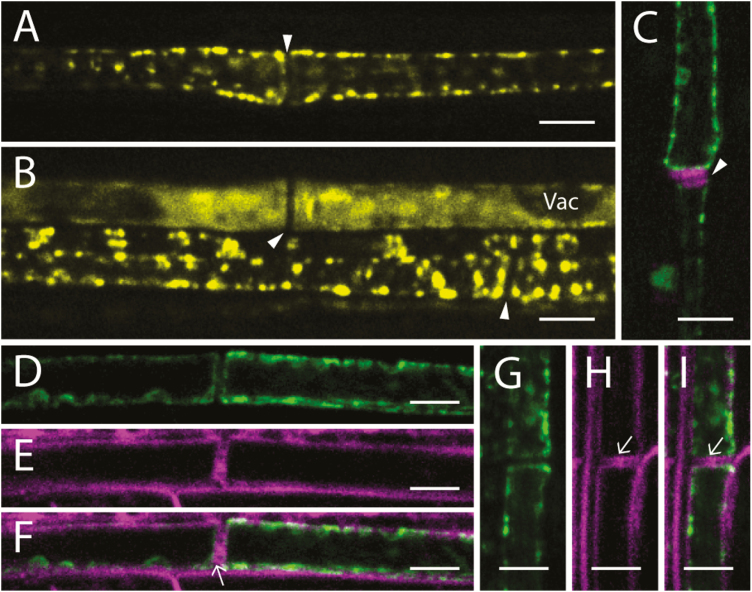
AtSuSy5–YFP and AtSuSy6–YFP were confined to the sieve elements of petioles. (A, B) Longitudinal sections of leaf petioles showing AtSuSy5 in mature sieve elements (A) and in immature sieve elements (B) containing vacuoles (Vac). (C) YFP labeling of AtSuSy6 (green) in mature sieve elements; the magenta signal indicates the sieve plates (white arrowhead) stained with aniline blue. (D–F) Longitudinal sections of leaf petioles showing the cytoplasmic localization of AtSuSy5. YFP signal of AtSuSy5 (green) is shown in (D), while the plasma membrane stained with FM4-64 (magenta) is shown in (E). Merged images of SuSy5–YFP and plasma membrane stained with FM4-64 are shown in (F). Plasmodesmata are indicated by an arrow. (G–I) Longitudinal sections of leaf petioles showing AtSuSy6–YFP (green) in (G) while the plasma membrane is labeled with FM4-64 (magenta) in (H). Merged images of SuSy6–YFP and plasma membrane stained with FM4-64 are shown in (I). Scale bars=5 μm.

In order to exclude the possibility that the four remaining SuSy proteins may be localized to the xylem and facilitate the production of UDP-glucose directly from sucrose to permit cellulose biosynthesis, C-terminal YFP fusion constructs of *SuSy2*, *SuSy3*, *SuSy5*, and *SuSy6* were generated (*SuSy2*_*pro*_*:: SuSy2*::*YFP*, *SuSy3*_*pro*_*::SuSy3*::*YFP*, *SuSy5*_*pro*_*::SuSy5*::*YFP*, and *SuSy6*_*pro*_*::SuSy6*::*YFP*), independently transformed into WT Arabidopsis, and analyzed by confocal microscopy. Similarly, cross-sections from the base to the top of an elongating stem clearly showed a phloem-specific localization of SuSy5 and SuSy6 (Supplementary Fig. S5A–F). Again, longitudinal sections of leaf petioles were examined to determine the subcellular localization of SuSy5 and SuSy6. Consistent with Barratt *et al.* (2009), SuSy5 and SuSy6 were only apparent in the sieve elements, including both immature sieve elements containing vacuoles and mature sieve elements that are fully functional (Fig. 5A–C). These observations were further confirmed by aniline blue staining of sieve plates (Fig. 5C). Interestingly, in the translocating mature sieve elements, YFP fusions appeared to be parietally positioned and largely accumulate as puncta, and were distributed along the entire sieve element (Fig. 5A, C). Moreover, both the SuSy5–YFP and SuSy6–YFP fusions were immobile, as shown by time-lapse video (Supplementary Movie S2). In contrast, fluorescence occurred uniformly in the cytoplasm when the sieve element was immature (Fig. 5B). Employing the styryl dye FM4-64, which specifically labels the plasma membrane (Bolte *et al.*, 2004), the precise localization of SuSy5 and SuSy6 was shown to be restricted to the inner side of the plasma membrane, indicating a cytoplasmic localization of these two proteins (Fig. 5D–I). This observation was most evident at the sieve plates, where the plasmodesmata were also stained by FM4-64. These observations provide additional evidence that SuSy proteins are not present in the developing xylem of Arabidopsis plants.

### SuSy2 and SuSy3 are highly expressed in developing seeds and leaf stomata

**Fig. 6.**
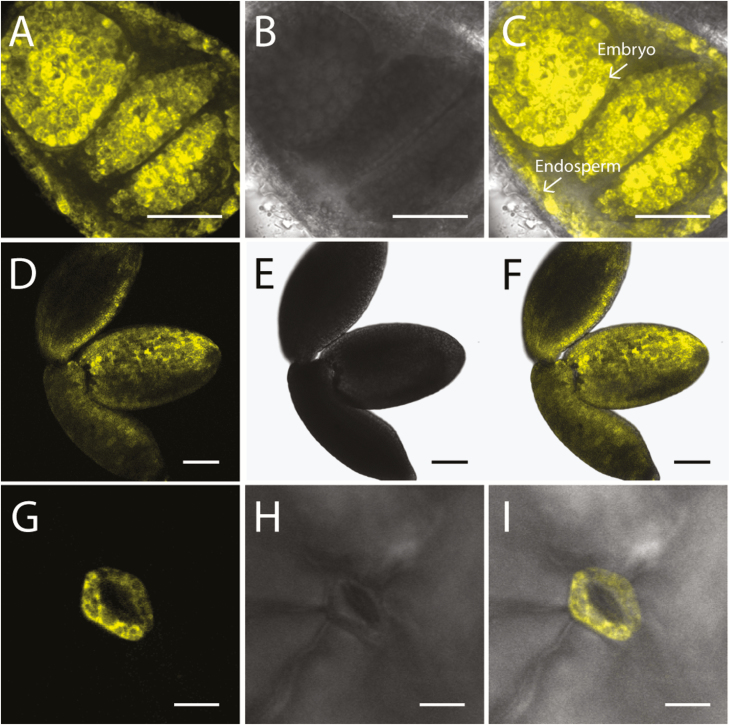
AtSuSy2–YFP and AtSuSy3–YFP were highly expressed in developing seeds and leaf stomata. (A–C) Cross-sections of Arabidopsis seeds at 13 DPA showing SuSy2 in the embryo and endosperm (arrows). (D–F) Intact embryo at 17 DPA showing that SuSy3–YFP fusions were present in abundance in embryo cells. (G–I) Leaf imaging showing that SuSy3 was detected in the guard cells of stomata. (A), (D), and (G) are YFP fluorescence panels, while (B), (E), and (H) represent bright field images, and (C), (F), and (I) are merged images of YFP fluorescence and bright field images. Scale bars=50 μm in (A–C), 100 μm in (D–F), and 10 μm in (G–I).

SuSy2 and SuSy3 were absent or undetectable in the vasculature of elongating stem (Supplementary Fig. S5G–L). Previous studies have shown that Arabidopsis SuSy2 and SuSy3 were induced in seeds, but barely detectable in other tissues (Bieniawska *et al.*, 2007; Nunez *et al*., 2008). Therefore, live-cell imaging was performed on the cross-sections of transgenic seed, and SuSy2 was apparent in the embryo and endosperm (Fig. 6A–C). YFP signal of SuSy2 was widely detected in embryo cells from 10 to 17 days post-anthesis (DPA), and then the signal faded quickly after 17 DPA (Supplementary Fig. S6). Consistent with previous data, our current work shows that SuSy2 activity was not detectable in the outer integuments of the seed coat, when the mucilage was secreted and secondary cell wall was deposited (Supplementary Fig. S7 C,D). In contrast, SuSy3 was highly expressed in embryo cells at 17 DPA and was also observed in the guard cells of leaf stomata (Fig. 6D–I). Images in higher resolution showed that SuSy2–YFP and SuSy3–YFP were confined to the cytoplasm of embryo cells (Supplementary Fig. S8).

## Discussion

Although the SuSy pathway has been generally considered the dominant route of sucrose catabolism in many plants, its essential role in Arabidopsis growth and development has been questioned by several studies (Bieniawska *et al.*, 2007; Barratt *et al.*, 2009). Thus, to gain further insights into the potential functional role(s) of SuSy in Arabidopsis, as well as to investigate its direct role in cellulose biosynthesis, we used live-cell imaging to localize all six SuSy isoforms under the control of their respective native promoters. High-resolution imaging of fluorescently tagged SuSy was carried out using a confocal microscope in several xylem-producing tissues including stems, leaves, siliques, and roots. Our results clearly demonstrated that most SuSy isoforms were exclusively expressed in the phloem, and none of the six SuSy was detectable in developing Arabidopsis xylem. SuSy1 and SuSy4 were specifically localized to phloem companion cells, where they are most probably involved in sucrose translocation.

Consistent with Bieniawska *et al.* (2007), *sus1/sus4* double mutant plants exhibited no obvious growth phenotype when grown in well-aerated conditions, with the exception of mild chlorosis, but showed significantly reduced growth rates and accumulation of soluble carbohydrate when subjected to flooding (Fig. 1; Supplementary Table S4). Similar phenomena were also apparent in maize mutants lacking SuSy activity, which showed root death only during anoxic growth conditions (Ricard *et al.*, 1998). It has been suggested that both SuSy1 and SuSy4 are necessary for plants to cope with anaerobic stress, as the transcript abundance of *SuSy1* and *SuSy4* dramatically increased when plants were subject to anaerobic environments (Baud *et al.*, 2004; Bieniawska *et al.*, 2007). Elevation of SuSy transcript abundance and activity in response to hypoxic conditions has also been reported in wheat (Albrecht and Mustroph, 2003) and potato (Biemelt *et al.*, 1999). This specific response has been proposed to be biologically beneficial under these conditions, as it offers an energetically more efficient means to supply UDP-glucose to biological processes: requiring a single enzymatic step (via SuSy), while sucrose hydrolysis via invertases requires several additional biochemical conversions for the generation of UDP-glucose (Canam *et al.*, 2006). Thus, under anaerobic stress and with limited ATP supply, the SuSy pathway may be favored in some plants. Evidence supporting this claim includes the rapid decrease in the INV:SuSy activity ratio when maize roots (Zeng *et al.*, 1999) and rice seedlings (Guglielminetti *et al.*, 1995) were exposed to low levels of oxygen. Moreover, leaf chlorosis has been reported to be associated with the inhibition of phloem loading (Zhang *et al.*, 2014). Consistent with these observations, in this study, leaf chlorosis was obvious in 6-week-old *sus1/sus4* plants and was more severe after flooding treatment, supporting a putative role for these two SuSy isoforms in phloem loading in Arabidopsis.

In support of this claim, our live-cell imaging data clearly show that SuSy1 and SuSy4 were exclusively localized to phloem companion cells, and were not present in the developing xylem of all the tissues examined (Figs 2, 3; Supplementary Fig. S1). Transcript results and western blot analysis indicate that secondary cell wall cellulose was still actively being synthesized in the elongating stem tissues employed for imaging (Fig. 4). In addition, our results show that neither the SuSy1–YFP nor the SuSy4–YFP fusion was detectable during secondary cell wall deposition in VND7-induced protoxylem tracheary elements (Supplementary Fig. S4). Instead, it was apparent that both SuSy1 and SuSy4 isoforms were actively streaming in the cytoplasm of companion cells of mature leaf petioles (Supplementary Movie S1). The current findings are consistent with the observed SuSy1 activity within phloem cells shown by promoter–GUS fusion studies (Martin *et al.*, 1993). Furthermore, companion cell-specific localization of SuSy has been reported in several species (Nolte and Koch, 1993; Fallahi *et al.*, 2008; Regmi *et al.*, 2016). Our current study uniquely examined the tissue and subcellular localization of all SuSy isoforms. These findings, therefore, provide insight into the potential role(s) of SuSy in energy-dependent sucrose loading. Companion cells are very metabolically active and distinguished by a dense cytoplasm containing numerous mitochondria, plastids, and free ribosomes (Cayla *et al.*, 2015), and communicate with sieve elements via plasmodesmata to supply energy and macromolecules. In Arabidopsis, sucrose is believed to be actively loaded into the phloem from the apoplast via a plasma membrane-localized AtSUC2 H^+^–sucrose symporter in the companion cells (Stadler and Sauer, 1996). This symporter has been proposed to be important not only for phloem loading in the sink tissues, but also for sucrose retrieval during long-distance transport (Truernit and Sauer, 1995; Stadler and Sauer, 1996; Gottwald *et al.*, 2000). Our data show that the distribution pattern of SuSy isoforms is similar to that of the AtSUC2 H^+^–sucrose symporter. In addition, we show that SuSy1 was highly expressed in the symplastic unloading zone at the end of the funiculus (Supplementary Fig. S3A–C). In Arabidopsis, sucrose unloading at the terminal ends of phloem has been reported to be mainly symplastic, as observed in anthers (Imlau *et al.*, 1999), developing seeds (Imlau *et al.*, 1999; Stadler *et al*., 2005*a*), and root tips (Stadler *et al*., 2005*b*). Though the exact function of SuSy in this passive diffusion mechanism is not clear, one possibility is that the sucrose concentration is modulated by SuSy, and may regulate the efficiency of symplastic unloading.

In contrast to the companion cell specificity of SuSy1 and SuSy4, we show that SuSy5 and SuSy6 were confined to phloem sieve elements (Fig. 5A–C), consistent with Barratt *et al.* (2009). However, our high-resolution imaging expands these findings to show that SuSy5 and SuSy6 occurred predominantly as immobile puncta at the margin of sieve tubes, and were not co-localized with the plasma membrane (Fig. 5D–I). These unique observations may be explained by the ultrastructure of a sieve element, which has lost its nuclei, ribosomes, and vacuole during maturation, but still contains smooth endoplasmic reticulum, plastids, and P-protein (Knoblauch and van Bel, 1998; Cayla *et al.*, 2015). These organelles are usually embedded in an amorphous ground matrix that attaches to the plasma membrane or P-protein filaments (Froelich *et al.*, 2011). Thus, we propose that SuSy5 and SuSy6 are likely to be trapped in this parietal protein layer (see lack of movement in Supplementary Movie S2) and play a role in cellular metabolism of sieve elements, such as controlling osmolyte levels. Sieve plates provide connections among adjacent sieve elements, and the callose lining of sieve plate pores is essential for normal phloem transport (Barratt *et al.*, 2011). SuSy5 and SuSy6 may therefore also be involved in callose synthesis, as double mutants displayed defects in callose lining of the sieve plates (Barratt *et al.*, 2009). Our subcellular localization supports these claims. In addition, via immunofluorescence, SuSy has been shown to co-localize with H^+^-ATPases in sieve elements of castor bean tumor (Wächter *et al.*, 2003). A sucrose transporter, AtSUC3, has previously been identified in the phloem sieve elements and proposed to be responsible for the retrieval of sucrose during phloem transport (Meyer *et al.*, 2004). Therefore, we postulate that SuSy5 and SuSy6 could be involved in providing energy for this sucrose retrieval.

Consistent with previous studies, SuSy2 and SuSy3 were not present or detectable in the vasculature of elongating stems, but were specifically and strongly expressed in developing seeds and leaf stomata (Fig. 6; Supplementary Figs S5G–L, S6; Bieniawska *et al.*, 2007; Fallahi *et al.*, 2008; Nunez *et al*., 2008). Our results show that SuSy2 activity was highly induced in the cytoplasm of embryo cells from 10 to 17 DPA (Supplementary Fig. S6), while SuSy3 was widely expressed throughout the embryo at 17 DPA (Supplementary Fig. S8). In addition, transiently decreased starch content and sucrose accumulation has been reported in the single knock outs of SuSy2 and SuSy3 (Angeles-Nunez and Tiessen, 2010), suggesting that SuSy2 and SuSy3 may have a specific role in embryo development. Furthermore, SuSy3 was shown to be present in the guard cells of leaf stomata. Daloso *et al.* (2016) have shown that the up-regulation of AtSuSy3 in tobacco plants led to increased stomatal aperture and conductance, transpiration rate, net photosynthesis rate, and plant growth. These findings suggest that SuSy3 may be specifically involved in guard cell metabolism/function. Moreover, based on the collective observations, we suggest that SuSy2 and SuSy3 are not likely to be associated with cellulose synthesis, but are important to seed development and stomatal metabolism.

No single fluorescently tagged Arabidopsis SuSy was detectable in the xylem of the organs examined, while most SuSy proteins were restricted to the phloem. These findings suggest that the bulk of cellulose biosynthesis in Arabidopsis does not use UDP-glucose channelled directly from particulate SuSy, but instead relies on the free pool of cytoplasmic UDP-glucose. Free UDP-glucose may be generated partially by the activity of SuSy, but is more likely to be formed via UDP-glucose pyrophosphorylase from glucose released from sucrose via INVs. This is consistent with the recent findings of Barnes and Anderson (2018), who showed that an Arabidopsis mutant lacking two cytosolic INV isoforms exhibited abnormal cellulose biosynthesis and significantly reduced UDP-glucose content. However, SuSy could still impact cellulose deposition indirectly, by modulating carbon allocation and sink strength. Thus, the current model in Arabidopsis, at the very least, should be reconsidered, and similar studies should be initiated in other species reporting the phloem specificity of SuSy. However, it is still possible that in some plant species the proposed association between SuSy and cellulose synthase exists in certain cell types, such as cotton fibers and developing poplar xylem, which have extremely high rates of cellulose synthesis. Consistent with this, in these systems, overexpression of SuSy genes has manifested in improved cell wall biosynthesis, resulting in elevated cellulose production (Coleman *et al.*, 2009; Xu *et al.*, 2012). However, this does not necessarily have to come from a direct interaction between SuSy and the cellulose synthase complex. In addition, Gerber *et al.* (2014) showed that deficient SuSy activity in developing wood of aspen (*Populus*) did not affect cellulose biosynthesis, but led to a decrease in wood density, which again suggests that SuSy might play a role in defining carbon partitioning into wood. Therefore, our results highlight the need to re-examine the intricacies of secondary cell wall development and more specifically cellulose biosynthesis in other species, especially those generating significant quantities of cellulose in a relatively short period of time.

In summary, we examined the precise subcellular localization of three phylogenetically related pairs of Arabidopsis SuSy proteins. Despite the high expression level of SuSy1 and SuSy4 in stem tissues as previous studies suggest, they were specifically localized to phloem companion cells, not to developing xylem, which implicates a predominant role in sucrose translocation, not direct cellulose biosynthesis. Furthermore, SuSy5 and SuSy6 were confined to the cytoplasm of sieve elements, and SuSy2 and SuSy3 were highly induced during seed development and only SuSy3 was found in leaf stomata. Our data contribute to a better understanding of the potential functionality of SuSy, and permit a more refined understanding of the potential roles for SuSy isoforms in cell wall development, and general growth and development in Arabidopsis.

## Supplementary data

Supplementary data are available at *JXB* online.

**Fig. S1.** Spatial localization of AtSuSy4.

**Fig. S2.** Phloem localization of AtSuSy1–YFP and AtSuSy4–YFP in the roots.

**Fig. S3.** Different spatial localization of AtSuSy1–YFP and AtSuSy4–YFP in developing seeds.

**Fig. S4.** YFP signal of SuSy1 and SuSy4 was not detected in the VND7-induced tracheary elements.

**Fig. S5.** Confocal images of AtSuSy2–YFP, AtSuSy3–YFP, AtSuSy5–YFP, and AtSuSy6–YFP in elongating inflorescence stems.

**Fig. S6.** Localization of AtSuSy2 in developing embryo of Arabidopsis.

**Fig. S7.** AtSuSy2–YFP was not detected in the epidermis of Arabidopsis seed coat.

**Fig. S8.** Subcellular localization of AtSuSy2–YFP and AtSuSy3–YFP in embryo cells.

**Movie S1.** Motility of SuSy4–YFP in the companion cells.

**Movie S2.** SuSy5–YFP was immobile in the sieve elements of petiole longitudinal sections.

**Table S1.** Primers employed for real-time PCR analysis.

**Table S2.** Primers employed for the amplification of SuSy-coding sequences.

**Table S3.** Primers used for amplification of SuSy promoter fragments.

**Table S4.** Growth phenotype of *sus1/sus4* mutant plants subjected to flooding.

erz539_suppl_Supplementary_Figures_S1-S8

erz539_suppl_Supplementary_Tables_S1-S4

erz539_suppl_Supplementary_Data

erz539_suppl_Supplementary_Video_S1

erz539_suppl_Supplementary_Video_S2
