# Supplementary material for: Arabidopsis sucrose synthase localization indicates a primary role in sucrose translocation in phloem
Source: J Exp Bot. 2019 Dec 5;71(6):1858–69. doi: 10.1093/jxb/erz539 (PMC7242074; doi:10.1093/jxb/erz539)
Supplement: erz539_suppl_Supplementary_Figures_S1-S8 [file erz539_suppl_supplementary_figures_s1-s8.pdf]

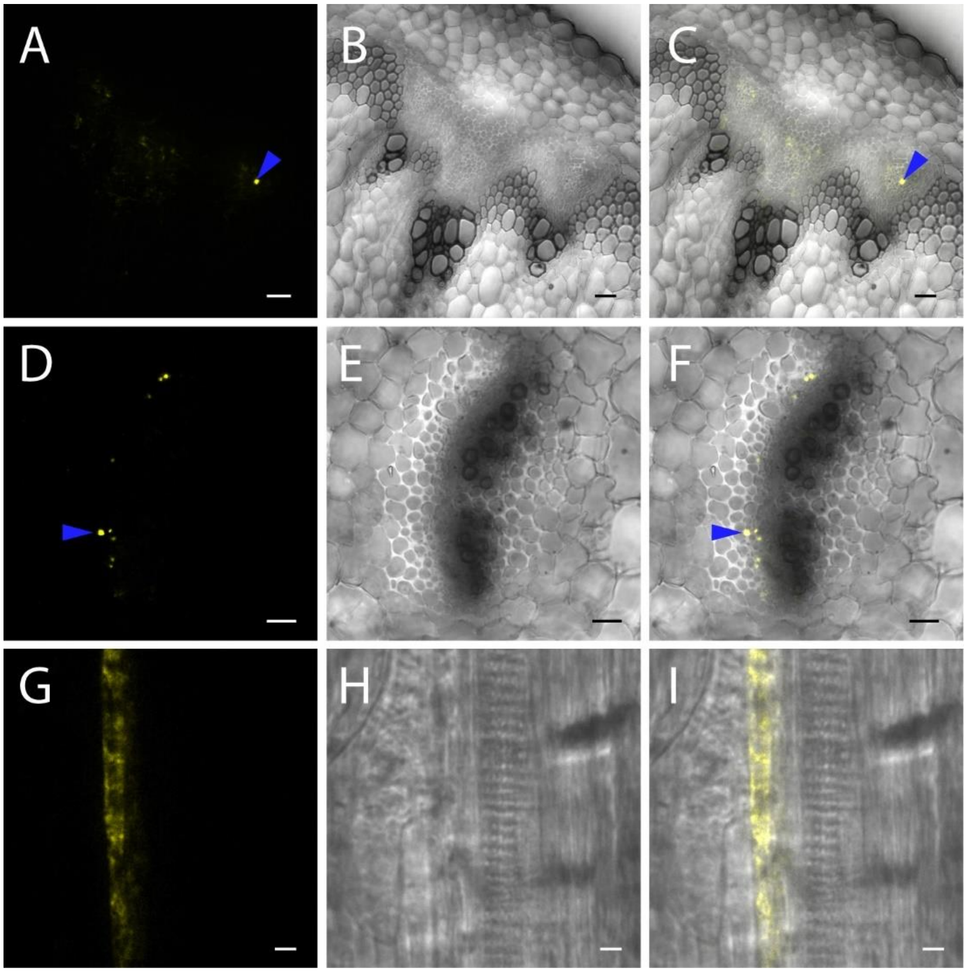

Fig. S1. AtSuSy4-YFP was localized to the phloem. (A-F) Images showing YFP signal of AtSuSy4 (blue arrowheads) in the phloem of stems (A-C) and petioles (D-F) cross-sections. (G-I) Longitudinal sections of silique wall showing that AtSuSy4-YFP was localized in phloem. A, D and G are YFP fluorescence panels, while B, E and H represent bright field images, and C, F and I are merged images of YFP fluorescence and bright field images. Bars=50 μm in A-F, 20 μm in G-I.

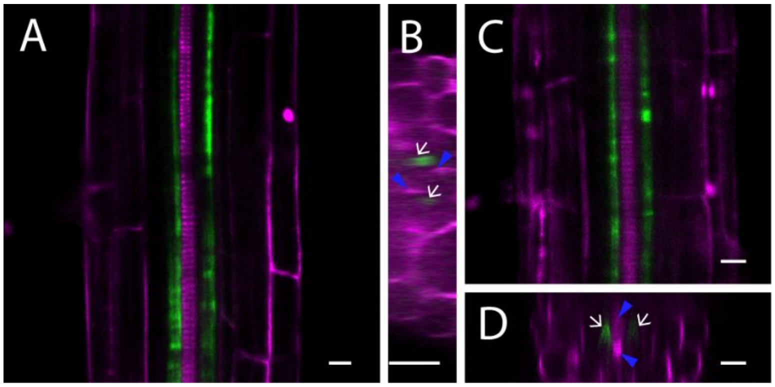

Fig. S2. Phloem localization of AtSuSy1-YFP and AtSuSy4-YFP in the roots. (A) YFP labelling of AtSuSy1 (green) in the phloem of 7-day-old *Arabidopsis* seedling roots. (B) Root sections showing AtSuSy1 confined to the phloem poles (arrows). Propidium iodide staining (magenta) was used to label protoxylem (arrowheads). (C) AtSuSy4-YFP (green) was localized to the phloem of seedling roots. (D) Root sections showing AtSuSy4 was confined to the phloem poles (arrows). Propidium iodide staining (magenta) was used to label protoxylem (arrowheads). Bars=10 μm.

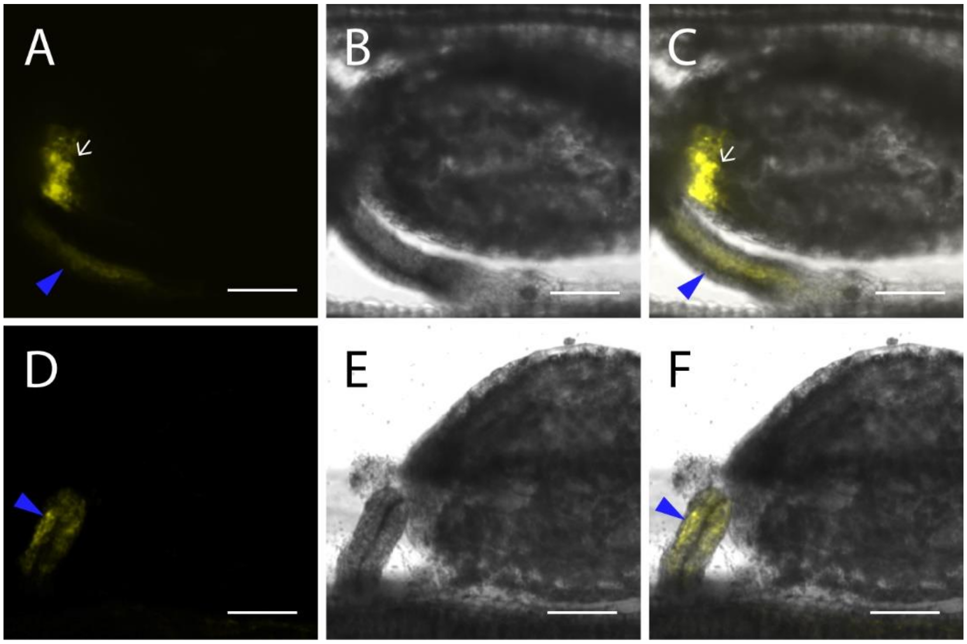

Fig. S3. Different spatial localization of AtSuSy1-YFP and AtSuSy4-YFP in developing seeds. (A-C) AtSuSy1-YFP fluorescence was detected in the funiculus (blue arrowheads) and unloading zone (white arrows) of developing seeds. (D-F) YFP signal of SuSy4 was detected in the funiculus of *Arabidopsis* seeds, as indicated by blue arrowheads. A and D are YFP fluorescence panels, while B and E represent bright field images, and C and F are merged images of YFP fluorescence and bright field images. Bars=50 μm.

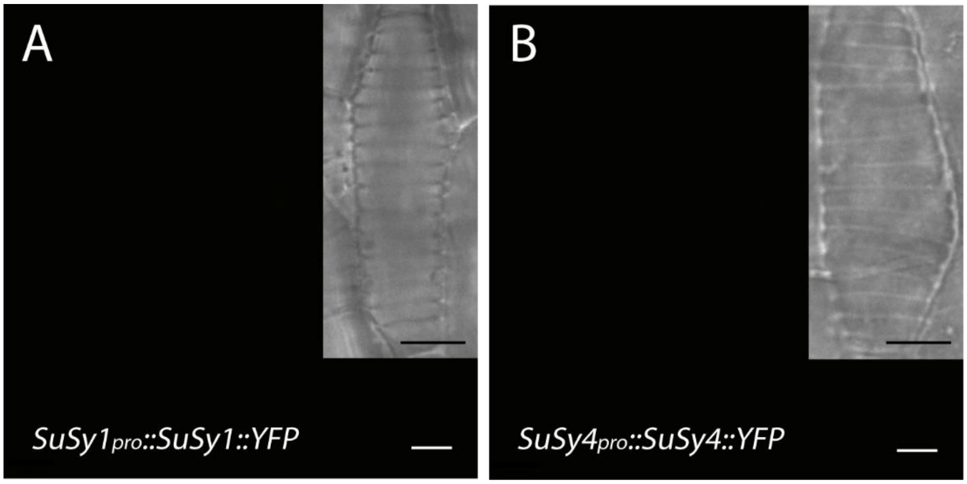

Fig. S4. Live-cell imaging of 7-day-old *Arabidopsis* seedlings showed that the YFP signal of SuSy1 and SuSy4 was not detected in the VND7-induced tracheary elements during all stages of secondary cell wall deposition. (A) AtSuSy1-YFP fluorescence was not detected in the transdifferentiated protoxylem tracheary elements cells induced with dexamethasone. (B) No YFP signal of AtSuSy4 was detectable in the transdifferentiated protoxylem tracheary elements cells following induction with dexamethasone. A and B are YFP fluorescence panels and inserted images are bright field images showing the morphology of transdifferentiated tracheary elements. Bars=10 μm.

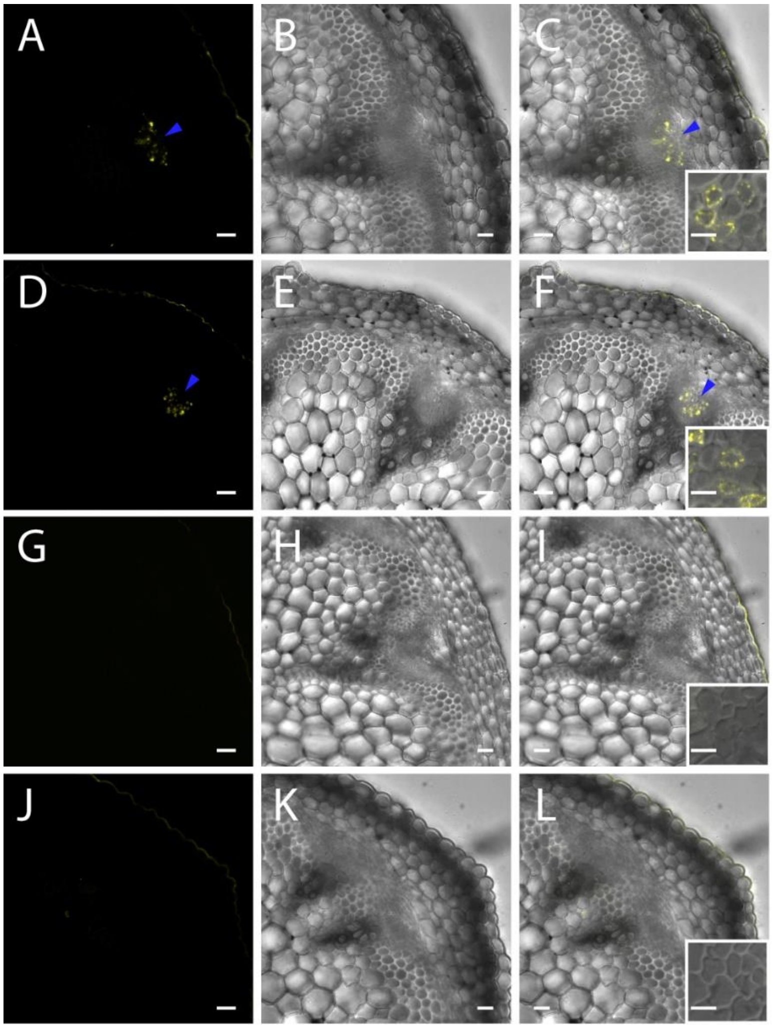

Fig. S5. Confocal images of AtSuSy2-YFP, AtSuSy3-YFP, AtSuSy5-YFP and AtSuSy6-YFP in the base of elongating inflorescence stems. (A-C) Cross-sections of inflorescence stem showing AtSuSy5-YFP specifically localized to the phloem (blue arrowheads). (D-F) Cross-sections of inflorescence stem showing AtSuSy6-YFP confined to the phloem (blue arrowheads). (G-I) AtSuSy2-YFP was not detectable in the vascular bundles of inflorescence stems. (J-L) No YFP signal of AtSuSy3 was apparent in the vascular bundles of stem cross-sections. Insert images are phloem cells shown at higher magnification and scale bars represent 5 μm. A, D, G and J are YFP fluorescence panels; B, E, H and K represent bright field images; C, F, I and L are merged images of YFP fluorescence and bright field images. Bars=50 μm.

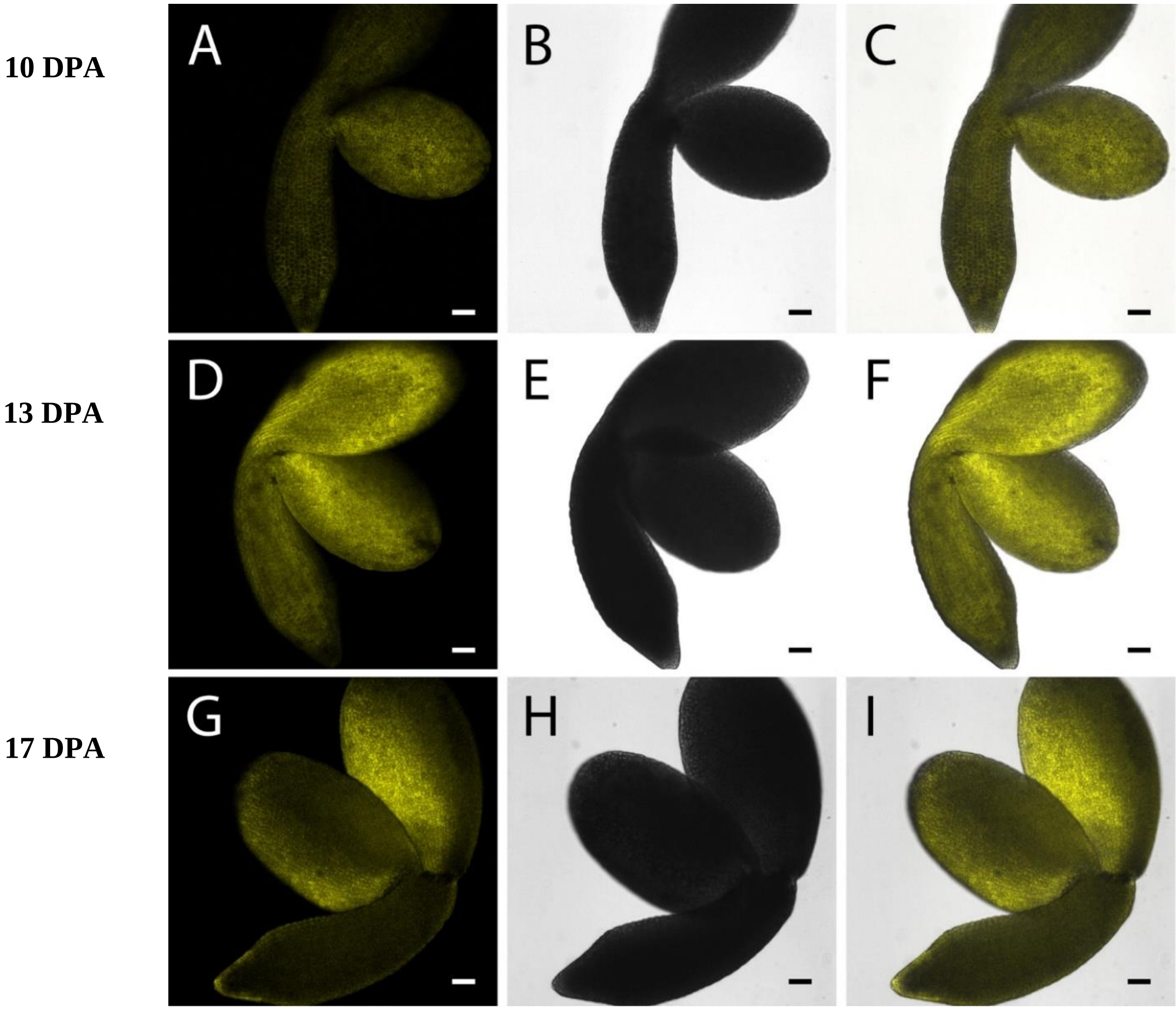

Fig. S6. AtSuSy2-YFP was clearly observed in the developing embryo of *Arabidopsis* seeds from 10 DPA to 17 DPA. A, D and G are YFP fluorescence panels, while B, E and H represent bright field images, and C, F and I are merged images of YFP fluorescence and bright field images. Bars=50 μm.

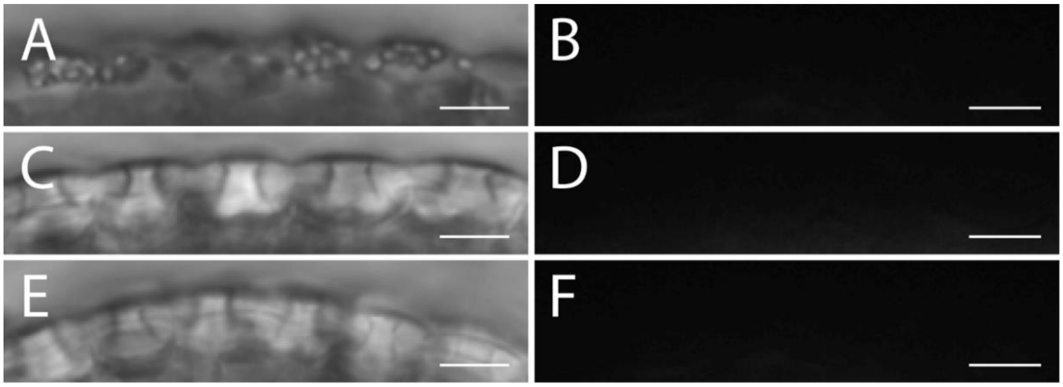

Fig. S7. AtSuSy2-YFP was not apparent in the epidermis of *Arabidopsis* seed coat from 7 DPA to 13 DPA. (A, B) When the starch-containing amyloplasts were accumulated in the epidermis (A), SuSy2-YFP fusions were not present in the cells of outer integuments at 7 DPA (B). (C, D) At 10 DPA, a large quantity of mucilage was synthesized and secreted, and the secondary cell wall was subsequently deposited (C). YFP fluorescence of SuSy2 was not detected in the epidermis of seed coat (D). (E, F) At the end of seed maturation, structure of epidermal cells was preserved by the mucilage and columella, SuSy2-YFP fusions were not detectable in epidermal cells. A, C and E are bright field images, while B, D and E represent YFP fluorescence panels. Scale bars=10 μm.

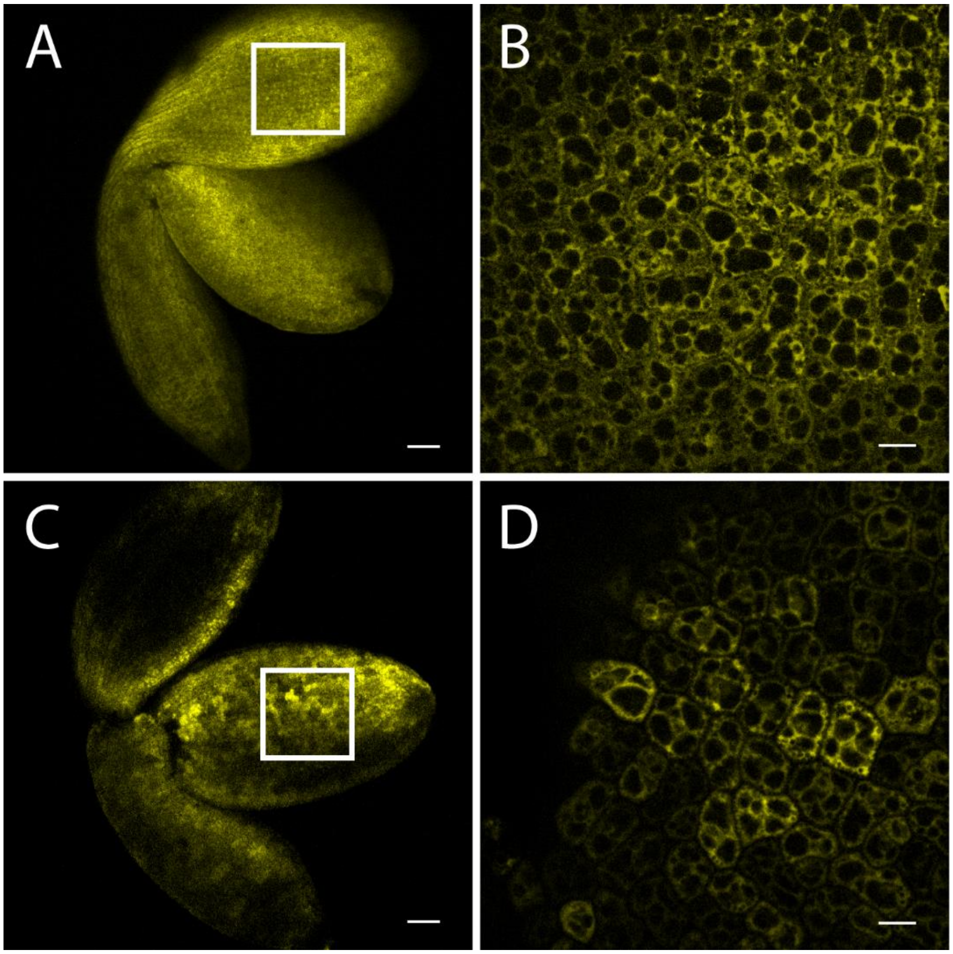

Fig. S8. AtSuSy2-YFP and AtSuSy3-YFP fusions were present in the cytoplasm of *Arabidopsis* embryo cells. (A) AtSuSy2-YFP fusions were highly induced in embryo at 13 DPA, as shown in Fig. S6D. (B) Areas indicated by the corresponding white box in panel A showing embryo cells at higher magnification. (C) YFP signal of AtSuSy3 was detected in abundance in embryo cells at 17 DPA. (D) Areas indicated by the corresponding white box in panel C showing that SuSy3-YFP was confined to the cytoplasm of embryo cells. Bars= 50 µm in A and C, 10 µm in B and D.
