# Supplementary material for: Arabidopsis sucrose synthase localization indicates a primary role in sucrose translocation in phloem
Source: J Exp Bot. 2019 Dec 5;71(6):1858–69. doi: 10.1093/jxb/erz539 (PMC7242074; doi:10.1093/jxb/erz539)
Supplement: erz539_suppl_Supplementary_Tables_S1-S4 [file erz539_suppl_supplementary_tables_s1-s4.pdf]

Movie S1.

Motility of AtSuSy4-YFP in the companion cells of longitudinal sections of leaf petioles. Time-lapse video showing that AtSuSy4-YFP fluorescence was streaming in the cytoplasm of companion cells. Movie was acquired at 5-second intervals for total 1:35 minutes. Bar=10 μm.

Movie S2.

AtSuSy5-YFP was shown to be immobile in the sieve elements of petiole longitudinal sections. Time-lapse video showing that AtSuSy5-YFP fluorescence was parietally localized in sieve elements and YFP signal was immobile as indicated by arrowheads. Movie was acquired at 5 seconds intervals for total 1:35 minutes. Bar=10 μm.

Table S1. Primers employed for real-time quantitative PCR analysis.

Gene	Orientation	Primer sequences (5′ to 3′)
SuSy1 (At5g20830)	Forward	TTGCCTGAACAAACCCGGAA
	Reverse	A CCTCACAGCTAGAGCAACCC
SuSy4 (At3g43190)	Forward	AAGGAATCGTTCGCAAATGG
	Reverse	TTTCAGCGGCAACATCCTC
CesA4 (At5g44030)	Forward	GTCTGTGGCGATGAGGTCAA
	Reverse	AGAGTGTTGCATTGAGGGCA
CesA7 (At5g17420)	Forward	GTGGTGTTCCTCCGTCATCA
	Reverse	CAGCTCAGTTCCCCACTCAG
CesA8 (At4g18780)	Forward	AGAGAACGCATTGGTGAGGG
	Reverse	CGCACGGCTTTGCTATTGTT
UBQ5 (At3g62250)	Forward	CAGCTCCACAGGTTGCGTTA
	Reverse	CAAGCCGAAGAAGATCAAGCACAAG

Table S2. Primers employed for the amplification of SuSy coding sequences.

Gene	Orientation	Primer sequences (5′ to 3′)
SuSy1 (At5g20830)	Forward	CACCATGGCAAACGCTGAACGTAT
	Reverse	ATCATCTTGTGCAAGAGGAAC
SuSy2 (At5g49190)	Forward	ATGCCGACTGGTAGGTTCGAGACTAT
	Reverse	GTTCTCATCTGTTGCCAGCGGGATTG
SuSy3 (At4g02280)	Forward	ATGGCAAACCCTAAGCTCACTAGGG
	Reverse	GTCATCGGCGGTTGAAGGAACAG
SuSy4 (At3g43190)	Forward	CACCATGGCAAACGCAGAA
	Reverse	CTCTTCATGAGCAAGAGGAACA
SuSy5 (At5g37180)	Forward	ATGGAAATGACATCTGGATCG
	Reverse	AGCACCAAACAACCTGAAACTC
SuSy6 (At1g73370*)*	Forward	ATGTCATCTTCATCTCAAGCTATGC
	Reverse	ATACTCTTGAGCCGAGTTAGCACC

Table S3. Primers used for the amplification of SuSy promoter fragments.

Gene	Promoter size	Restriction sites added	Orientation	Primer sequences (5′ to 3′)
SuSy1	2090 bp	*Cla*I	Forward	TCTATCGATAAGTCAAGGTTAATC
		*Avr*II (*Xmaj*I)	Reverse	GCGCCT AGGTGATCCAAAAAAGA
SuSy2	600 bp	*Pme*I (*Mss*I)	Forward	CTCGTTTAAACCGACTAAAGAATTCTG
		*Spe*I	Reverse	CGCACTAGTGATTTTTTCTCAGAGG
SuSy3	1394 bp	*Pme*I	Forward	CTCGTTTAAACCCGGATTGCCTCTATA
		*Spe*I	Reverse	GCGACTAGTGAATATTCAGATGATCA
SuSy4	1959 bp	*Cla*I	Forward	GTGATCGATTTCTTCAACAAAGCCCTTCA
		*Avr*II (*Xmaj*I)	Reverse	CCTAGGTTCAAACACAATCACAAAGC
SuSy5	1731 bp	*Sac*I	Forward	CTAGAGCTCAATGCTCATTGCTCG
		*Spe*I	Reverse	GCGACTAGTTGTGTTATGTACCTTG
SuSy6	686 bp	*Pme*I	Forward	CTCGTTTAAACACACAACAACGAAAGAG
		*Spe*I	Reverse	CGCACTAGTAGAAACAACTGAAGATTC

AtSuSy1 promoter fragment (2090bp):

atcgatAAGTCAAGGTTAATCAAGAAGTTAGGACTAGTTAATTGAACTAAAGAAAGGAAAAGAAAAAAA
GATTTGTTAAAACTGGAATTAATTATTTTCTTGAAAAGGTCAAAAAGGAAAACGTGGGATATATAGTT
GCAGCTTATTCATCAAACAGTTACAAGTTTTTTATTAAAAATTAGCAAAAACAAAACAAAAAAAACAA

CACATAACTTTTACAATTTATAATATAAATAAATAAACTTTTGGGTTGAAAAGTTAATTTAACATTAAT
ATTTAAATATTTTGAATTGCAAAATATTTGAGAATTAATAAAACTTTTTTTTTGCATTTATTGGAAATTA
AATATAACCTTAAACTAATACTTTAGATCATTTTCATATTTAGTCAAAATTATTCAACAAACTTTTATTA
CAATGATTCATTTTAATTGTATCAAACAAAATTTCCATAGAGTCAAACTTATAATTTATATGTTTAAAA
GCTGTATTTTTTTTTACTTTGGTTTTAAATATGATATTTGTGTGTAATTAATTTTTCTTGTTTGTCAAGTA
TTATTAAGATAGAGAGATATTATTTCATATCATCAAATGTAAATGTCTTGTTAATCCAATTATCCAAAG
ATCAGTGACATATCCGATATATCTATTCTATTTAAAGGAAAAAAACATTTAGTGATTGTTTTATTATAA
CATTTAAGGAAAATGTACACTATTCAGAACTTTCCTATTTTCCTAAAACACCCTTTTCCTTGGACTTTTA
AATCCATTATATTTAGTCTTCTGGGTTTGGAGCCCAAGAAATAAAACGTAGAAGGAAACAGAGGGAA
GTAAATGATTCATATCAGCTTACCAAAATTTGTATCAAGCTTTAAAAATTCGTCTCTCTATAAATCTGA
AACATATTGAACATTAACCTCCACATCTCACTCAAAACCACTTCTCTATACTCCAGGTTCATTTCTTCTT
CTTTAATCTTTAAAGTTTGGTTTAAAGTTTTTCTGATACATTTAGTTTAATCATTTTTCGTTCTTATGATA
GTTAACGAAACAAGTTTTTGTGTTTATTTTTCTGACAAAAAAGCAGAAAAATAAGATGAATCAAACAA
CATCTTGTTCTTTTTCTTAGAAAATTCTTGCCTGAAAAATCTCTGGTTAGTAAAAAAACACCAAAACGT
TTTTATATTCTAAAGAATCAGTCGTGATTTGTATTCTATTTTATCGCTTTTTAATTTTTTTAGAGGAATTT
TCTCATAGAGAATTTTCCAGCTGTGTTGCAGAAAACAAAAGAATGTCACGTCCAACATTTTCTTTAATT
TAGTAAACATTTTCGGAAAAATTCTTTTGCATTATGAACCATTTTCTCCCGATAATAAAACTCCAAATA
AGGAATCCACTATAGAGACTTTTGACTGTTCAAGAAACTCAAAATATATGTTTAGTTTTCCCTTTACAA
TCATAATTTCTTTTAATGTACTGTATGACGTGTACCAGGATTCTTATTCTTGTCTTTATTTTACATGTCTG
CATCCAACTTTTTTATATGTCACACACTTGGAATTAAATTTGACGAAGATGTTTTGTAACTTGTATGGA
TTCCTAAACACCATTCCTTTAAAATGGATGGTGGATAGGAAACGTTTAACCAAAAAACTAATGAATTT
GATGGATAAGATTGTTCTTTAAACATCAATTTCATTTTGTAAAACAATGTCCTATGTCGGCTAGAAAAT
ATATTAGTCAAAAACGAATTTTGGTTTCGCCATGTGTACATGTAGAGGTCAAGTACGTGAATATAATA
ACAACCTCTAGCTCTTAGCTCTACTGCTCTAGGCTTGTTTCGTAAAACCTTGGAAGCTTGAAGGCTAAA
AGCGTAAAAGAAAAGTCATGTTTTCAATAAATTTCTTGACTTTTAGTTATAATATCTTGACCTTTGTTAT
AATATCTAACCCATAGTTTTTCTTTATATATAATGTGAATATGGAAAACACAAGACTTCATCTTATATT
CATCAATCTTATTCTTATTTTCTCTCTGCAGTTTTCAAAAACTTTCTCACTGTTATCTGCGTCTCTTTTTT
GGATCAcctagg

AtSuSy2 promoter fragment (600bp):

CGACTAAAGAATTCTGATTTAATTTTGTGGTTTATATGTTGAGTTAACTGTTAAGAGAGTTTTATTTTGT
AATAGGTGTATCAGTCAATAAACAATCTTTGTATCAACCAAATGTAATTTTTCTCGTTAATTCGATTTC
AGAGTTTTTACTTTAAGATAAACAAACTCTTTCACACATCATTTAATGAAAGTGGAGAAGCTTAAAAA
ACAAACAAAGAAACTGATCCATTTTTGGCGGGTCTTCTTCTACTCTTATTCATATGTGTTAACGAACTA
TAGCGTAAAATTCAGAGCAAGCGATCTCCGATTTGAACGTGGCTATCACCGGAGGCCCACCACTACGG
GCGATACGCTCTAAGTGAGGATTAAAGTGCTCTGGTGGTGACGTTGAAGAAACTCGCCCATGGTTTTT
GTTATCTCTGCAGCCAAGTGTCGTTCTTTCTTCGCCACTTCTCATCAAGCTACAGTGAATTTAAAAATG

GCGTCTTTCTTTGATCTCGTATACATAAGCTGGATTGGTTTCTTAAACAAATTCCTCTCCTTTTGGGTCT
TCTGGGTTTGCCTTGTAAGTGTTTGTGTTTTTGCCTCTGAGAAAAAATC

AtSuSy3 promoter fragment (1394bp):

CCGGATTGCCTCTATACATATGATAAAGTTCTAACAAGAAACGGGTCATCTCGTCTGACCAAATAATTT
TCTAATCGAGAAAACAAATCAAAACATGTATTCAATCAAAAAACTCAAAAAAATACAAAATAAATAA
AATGACGATGCCATAAAATACTCAGAGAATTACACTAGCTAATGAATGGATGAAAGGATAGATCATA
CTTACGTTTGATTCTTGATTTTGATTTTGATTTTCGTTAGTTTGGGTAGATGCCATAATTGAGAGAATAG
GGTTTCAATATATTGATTAGGATTTTCTGATAAGAATAAGATATATAGGGTTTGGGTGGAAGACATAA
TTATTTTAGTTAATTAAAAAAAAAAAATTAACCTAACCGCTACCGCCCGCAACCGCAAACGCTTGCGGG
AAGGAACTTTTAAAATATGGCGATTTCGAGCGGTCCAAAGCGGTATCTAACGGTTTTTATGATTGGTGT
CAAACGCTAACAACTGCTACCACCCGCAAACGCAGCGTTTGCGGGTGACAGAGGGAGAACCAATCAA
GCTCTAAGACTAATCGGACCAATTTAGCAACTTGTGTGCTTCGATGTGTTGGACTGGGCTAGATTGGGC
CCAAATAGCCTGTTTTAACATTCCCAAAGATCCAAACAATTCCTTCTTCTTTCTTCCGAACAATTCAAT
GTAGATCCGAGCGAATCTCCTAAATTACTAAAATGCACAATTGTGCTCAGCTTACCAAAAAGGCCCAA
AAGACCAAAACATATTCTGATTTTTTCTCTTTAGATACAATAAAAGTAATTTATATAGATTAGAGGCCC
AATAAGGTTCACGTAGGCCCAAACATATTAAAAGTAACTCAGAAGAGATCCATCAAAATTTTGATTCA
CACATCTGTTTAGGAATTAAAAATATTATTTGATCGGTTATTCATTCCTTTTCATGAAATCATGCAAAA
AATCAAAAATCATTTTTTTCTCTAGAAACTACGTGGCGAGAAAGCAGAGCACCAGTTGTCTTCTTGCTC
TGATTATCTCGTTGAAACCGCTTTCAAAGCAGAGCAAAAGAGACGACACCGGAGCCTCCACTGCTTTA
CTTTTCCTTTAAACTGTGACTGCTTTCATTTATATAATAAAATACATACACTCTCAGAGTCACATGTACT
CTCCTCTAACATAAACACGTCACTTGTAGCGAAAACAGTATCAAGAAAAAGAGAAGATCAAACACGT
CTTCTTTTCTCTCTCTCTCTTTGTCGCCTAAAATTCCAGAATCACTCTGCTTTTTACCCTTTTAATCAATG
ATTTTTCCTTTTAGTAGCAATCGTTGGTGATTCGAAAAACCAAACTTTTCTCGGACTAGGATTCTAGGG
TTTTAGTGATCATCTGAATATTC

AtSuSy4 promoter fragment (1959bp):

atcgatTTCTTCAACAAAGCCCTTCACGGCCCCACTGCCCATACACACAAACAAGTGACAAGCAATTTTGA
ATCCATTTGATTAAATTTAATATCTTAAGCAAATTAAAATAACAAAGAAAACAAAAACTAGCTTGCTC
GTGGTAGTCTTGAAACCGTTGTTTTGTATGCCCATAGTTTACTGATGTTATTTTCTTTGGTTACTTTACT
TATTAGTTTATTGACAAATTTGACATTATGATTACAATAGTGAAGCTTGGTTAATTAAATTACTTTATT
GACATAATTATTATATATAAAATAATGAAGAAATTGTATGTTTTACACGCAGTACTCGATAACCAATA
GAATTCTTTTTCTGTGGATCAGTATTGAGTATTGACCCGTGGGTATTGACCAACTACTTACAATTATTA
TAACTTAAAACAAAATTGTATCTCTCTAAACAAAGCATAATTAGTATTAAAGAAAACCAAGTTGTCGA
AGAGAAGAGAGACTAGTATAAAATGATCCAACAAAGCTATTCAAATTCTTCACACTTCACTACTTATC
ATTTCTCTTCTTCATACTTTTCTCAAAATTTGCAATAGCTCCCTCCATCTTTCGCTTCATTCACTACATAT
TTCTAGTGACCTCCTTCGCCAGTGACTTTCCGTAGCAATTTAAGGTAATCTATTCTTGTAATGTATTATA

ATGAGAGGTTATAAGGTTTCAATGATTAGTTTAGTTTATGAAAATTTTTAATGCTCTGAATGTTTATGA
CATGCTAAACATTAGAACCTTCAAATATTTTTAGTTGAGTATGGTTAAAAAAGGTTTCACTGCTAATTA
ATTGATTTTTTTTTGGAAAAGTAAATCAATGGATTTTTGATTTCATTTTTTTTTATCTAGCAACACTAAA
AATAGAATTATATACTCGGACATTTTTCCGACACATGTAACATCGCTTAGCCTAAACTAAACTATTTAA
CTTTTTGTTGATTGGTGGTAAGAGGAAATTAAAAGCTTTTTAGAGGGAATATGCTTTGAAAGATTCTTT
AATAGTGTCATTTACTTTAGGATAAGAATAAAATTAGCATTGTCGTAAAAAGCATGCAACAAACACAT
TATAAACTTTTCTTCTTCTAAGAAAATAATTCAATTTAACAATTTAAGTTATTCGCTAATATATATATAT
ATATATTTTACAAAATTTTCAGCTAACAAATCATGTTTTCATTTTTTTGTTTTTGTTAAATATGATAATC
TTTCAATATATTTACTTTAGCTACTTTACGTTCCACAAAATATAAAATATTTCTTTTAATCAACAATAGA
TGTAAGATTTGATACCAAAAAATTTCTTGTTCATCAAGACAAATTAATGATTTATTTATATTTATTAATT
AATATTAATTTTTCTTGATCTATGTGATAAAATAAAAAAGATCTTTTTTATTTTAGATATTTTAGAATGA
TTCAGAAAATAAAACCAATAGCGTATATTCTGTTCTACTCGTCTTCTAATTTTGAAAATAATATTTTCA
CAGAGAATTTTCCAGCTATATGTCGAAATCTTTTGTATTTTTTTTTGCTATAAAAGAAAATTCGTTGAAT
TACTTTAATTCATGGTTTGATTTATCTTTTCCTCCATTACTTATTTGAGAAGCCATAAACACCGCTGCTT
TGAAATGGATACTGGATGCAAAATGTTTAAAGAAACTTAAATTGGACAGAATAAAGATGATTCTTTCA
ATATTTTATGTTGGAAAATATATATAATCAATTAATCATCTAACTTTAAAAAGTTAAGATAATTTTTGT
ATGATGGAATTTAACTAACTTTTTTCATAAGTCATAATCAATTTTTCCTTATATAGGCAAGCATTTTGA
ATTAACCTCTCTCCATTATCTTACTACATTTCTTCATCTTCTTATTCTCTGCAGTTTCAAAAGCTTTGTGA
TTGTGTTTGAAcctagg

AtSuSy5 promoter fragment (1731bp):

AATGCTCATTGCTCGTAGTTTAAAGCCCACATAAATTCTCTAGTAATCAATTGGAAGGATGATAAAGA
AATAGTTTGAGTATAAGGAAGATGTTTAATTGAATTTAAGTTTGGTTTGGAATAAGAGCTTCGGAGAA
ATGTACATAAGTTTTCTCAAAGAAGTAAAGATCTTACATAACGATGACATGTAAACATTTGAGCTTCA
AATACAATGATCATTATATTGTTTTGCAGATTGGCGATTGCATTGAATTAGAAACCAAAAAAAAATAT
ATATATATATATATGTTCTTTACTTCATTTGTATGGTCCACTTTTAGATCCTTTACTTCATCGTGCTCTCT
ACTTTTGGAATTTCCTTTGCTTTTCTCCTCTTCATCTACTACATAGAATCAAAAATGAAAACATCTTTTT
TTTACTCAATATTTACTCGTACTGACCTTGTCTTTTAATCTCTTTGTATTACTAAGTTTATGCTTCATGTG
ACAAAAAAAAAACAAAGATATAATAGACTGCCTATTTCTGTATATTTTCAGGTGATCATATTCTTATTT
CAGGTGAAAGAGAAGGAGAAACACAATTTTCAACCACAAAAAATACTTATCCGTAATTCTTATATTCG
ACAAAATATTTGTTACACTACCACTAATCTGATAATCATATATTACAGTTTTTGTTTTTTGGGTATGAAT
CATATATTATTGTTTTTAGGTTAATGTTACGACATCTAAAACCTAAAAAGCATATTGGATATATACTAA
TGGATCATTTGTCTAGTTCAATATTTTCTCAAAAAAATATGATTTGTGTGAATCTTGGTGGATTTTAAC
ATATGTAACTAAGCAAAATTTTGCAGCAATTTAGTGAGATAATTAGAAAACACTAAAACTTTGAATTG
ATATCTCGCTTCACATTCAAATGTGAAAACATATATTTTTTACTATTTAAAACATTAATCGAATTTACA
CGTTTTCAACTGAAAAATAATTTCATTCTATGCTATAAATTAAACTTGTCGAAAATATAGCTAGGTACA
ACGAAACATCACAAATATACATAAAGAAAAAATCCCAGAAAAAAACATTTTGAAAGTTTTTAATGTG

ATTTTCCCTATACTAAATTAATTTAGCATTTAATGATTGTTTTGTAGGAATTTTATCTAGCTAAAATGTT
CTTACCTAGTAGTTAGAAAAGCATACTAGCTGGATCACTTTTGAAAATGTAATTTTTTTGGTAAGCGTT
ATGAATGGTAGAAATTAGTTGAGTTATGTTTTCGAAACATAAATATTAATTTGTAAGGGTGATTCAACT
AAATTAGTTTATTAGTTACATTGATTAATATGTGTGAATATAATGAACCATAACTATGAAAACATCCCA
GCGAATAAAAATGAGAAAATTTGATCCCAAATTACAAGTAGGTGAGGGCAAGTTTACCCTGACTAAGT
TACGTACCGTGATTATAATTCTCTCATGTGGCAAGATATTTATTTGGTTAAGTCGTTCTAACAAAGAAG
AGAAAGCCAAATTAATTAGTTGGAGGACAAAACATGACCTTGCTAAAACGAAAGGTATATGACCCAC
ACACAATAACATATTCATAGAGATATGTCGTATCTTACCATAGTTCTCCTATAAAAGTAGACAAAGCT
GCCATTACGAAACCAATCAAAACCAACGCGAAAACCTGCGTTTTATTCTTCTTGTAATTGGTCTCAAGG
TACATAACACA

AtSuSy6 promoter fragment (686bp):

ACACAACAACGAAAGAGTCTAACCGGCTCAAAACCGGTTTGAGTAGCTAGCATATCGATATTCGGTCC
GATATCCGGTATGTTTCTCTGGCCGCTTGTTATATTCCGGTACATAATGAAAATCTCAACAAAATTAGT
TCAGTGTAACCATATGATTAATCAAGTGATGACTTAATTTCCTAGACTATAAATTGATCAACTTGAATC
TTTTTTCCAATTGGTACTAATTAATGGATAATTAGTACTGTATAATGATATAATTATTATCTTGCTATAA
AGTAATTTAGTATCATTGTCCACAGGGACCACAATCTTAAGTAAAGTATCCATCAAAGAGGGGGAAAA
GGAGCATTCTTTTTGCACCCATACCTTTTATGTTATTAACACATGAACCCCAAGAAAAGCAAAAGGTA
CACAAAATCTGATTTTGCTTCAAACAAAATGAAAATCACGCAATATTCTTTTTTTGTTAACTTGCTTGC
ACACTTTCTTTTCCCCACTTTTATTTCTAACGAATAATCTTCTCCCCCATCCTTCTCCTTCTACTCCTGTG
AGTCCTGTCCCCTTCACAGCATATTATTGTGTATCTTTATATATAAAATGTGGATTAAACCATGTTTCA
ACAAACTACTTCATATCTCAATCATTCAATATTCTTGAAGAGACTTTGAATCTTCAGTTGTTTCT

Table S4. Partial complementation of *sus1/sus4* mutant phenotype by SuSy1- or SuSy4-YFP fusion constructs. 4-week-old WT plants, *sus1/sus4* double mutants, and two independently transformed transgenic SuSy1 and SuSy4 lines subjected to flooding by maintaining degassed water above the surface of soil for 5 consecutive days. The control plants represent plants without flooding and were watered when necessary. Rosette leaf sizes were measured on the first and 5th day of growth. Mean (SE) values were calculated from at least 3 individual plants per line. Asterisks indicate significant difference (* p-value < 0.05, ** p-value < 0.01) of rosette size gain (Column A and Column B) using a Benjamini-Hochberg corrected Student *t*-test.

Line	Flooding			Control			
	Rosette size on the first day	Rosette size after 5 days	Size gain (A)	Rosette size on the first day	Rosette size after 5 days	Size gain (B)	% reduction in rosette size gain (1-A/B)×100
WT	3.24(0.08)	4.40(0.10)	1.16(0.09)	3.06(0.14)	4.27(0.13)	1.21(0.07)	4.71
sus1/sus4	2.90(0.11)	3.34(0.09)	0.44(0.06)**	2.80(0.26)	3.90(0.14)	1.10(0.12)	60.23
$SuSy1_{pro}SuSy1::YFP1$	2.96(0.16)	3.66(0.09)	0.70(0.12)**	2.97(0.37)	3.93(0.44)	0.97(0.13)	27.59
$SuSy1_{pro}SuSy1::YFP2$	2.50(0.15)	3.13(0.15)	0.63(0.09)**	2.65(0.23)	4.00(0.07)	1.35(0.14)	53.09
$SuSy4_{pro}SuSy4::YFP1$	3.15(0.06)	3.88(0.13)	0.73(0.10)**	2.60(0.11)	3.53(0.05)	0.93(0.14)	21.62
$SuSy4_{pro}SuSy4::YFP2$	3.10(0.17)	4.03(0.22)	0.93(0.12)	2.68(0.13)	3.70(0.25)	1.03(0.19)	9.76
