# Supplementary material for: Arabidopsis sucrose synthase localization indicates a primary role in sucrose translocation in phloem
Source: J Exp Bot. 2019 Dec 5;71(6):1858–69. doi: 10.1093/jxb/erz539 (PMC7242074; doi:10.1093/jxb/erz539)
Supplement: erz539_suppl_Supplementary_Data [file erz539_suppl_supplementary_data.docx]

**Supplementary data**

**Fig. S1.** Spatial localization of AtSuSy4.

**Fig. S2.** Phloem localization of AtSuSy1-YFP and AtSuSy4-YFP in the roots.

**Fig.** **S3.** Different spatial localization of AtSuSy1-YFP and AtSuSy4-YFP in developing seeds.

**Fig. S4.** YFP signal of SuSy1 and SuSy4 was not detected in the VND7-induced tracheary elements.

**Fig. S5.** Confocal images of AtSuSy2-YFP, AtSuSy3-YFP, AtSuSy5-YFP and AtSuSy6-YFP in elongating inflorescence stems.

**Fig. S6.** Localization of AtSuSy2 in developing embryo of *Arabidopsis*.

**Fig. S7.** AtSuSy2-YFP was not detected in the epidermis of *Arabidopsis* seed coat.

**Fig. S8.** Subcellular localization of AtSuSy2-YFP and AtSuSy3-YFP in embryo cells.

**Movie S1.** Motility of SuSy4-YFP in the companion cells.

**Movie S2.** SuSy5-YFP was immobile in the sieve elements of petiole longitudinal sections.

Table S1. Primers employed for real-time PCR analysis.

**Table S2.** Primers employed for the amplification of SuSy coding sequences.

**Table S3.** Primers used for amplification of SuSy promoter fragments.

**Table S4.** Growth phenotype of *sus1/sus4* mutant plants subjected to flooding.
